# Tobacco Use Among Middle and High School Students in Pennsylvania

**DOI:** 10.5888/pcd15.170289

**Published:** 2018-02-01

**Authors:** Sophia I. Allen, Jonathan Foulds, Emily Wasserman, Susan Veldheer, Shari Hrabovsky, Jessica Yingst, Guodong Liu

**Affiliations:** 1 Department of Public Health Sciences, Penn State Tobacco Center of Regulatory Science, Pennsylvania State University College of Medicine, Hershey, Pennsylvania; 2 Center for Applied Studies in Health Economics, Pennsylvania State University College of Medicine, Hershey Medical Center, Hershey, Pennsylvania

## Abstract

We analyzed data from the 2014–2015 Pennsylvania Youth Tobacco Survey to determine prevalence of tobacco use among middle and high school students. For the first time, questions on current (past 30-day) use of electronic cigarettes (e-cigarettes) were included in the survey. For current use, e-cigarettes were the most commonly used tobacco product among middle school students (2.3%; 95% confidence interval [CI], 1.4%–3.2%), and cigarettes were the most commonly used tobacco product among high school students (11.0%; 95% CI, 8.1%–13.8%). Given the changing landscape of tobacco products, collection of comprehensive data on tobacco use, including frequency, is important for monitoring behaviors among adolescents.

## Objective

Adolescents who initiate smoking and continue as adults are at high risk for developing serious health problems (1). Smoking harms brain development in adolescents and leads to sustained tobacco product use and addiction (1). Although tobacco use among adolescents in Pennsylvania has declined, the current and changing rate of electronic cigarette (e-cigarette) use is unknown (2). The Pennsylvania Department of Health’s comprehensive tobacco control program began monitoring e-cigarette use with the administration of the 2014–2015 Youth Tobacco Survey (YTS). This study described the prevalence of tobacco use among adolescents and assessed differences by demographic characteristics.

## Methods

Public middle schools (grades 6–8) and high schools (grades 9–12) were systematically selected to participate in the Pennsylvania YTS through a cross-sectional, 2-stage cluster sampling design to produce a representative sample of Pennsylvania students. Participants were 2,668 students from 72 middle schools and 2,017 students from 63 high schools. Overall response rates for survey completion were 74.5% (middle schools) and 64.7% (high schools). The methodology used for the sampling design is explained elsewhere (3). Demographic characteristics of interest were sex, race/ethnicity (non-Hispanic white, non-Hispanic black, Hispanic, and non-Hispanic other), and grade. Parental permission procedures (active and passive consent) were followed before survey administration. The Penn State Hershey Institutional Review Board determined that because the study involved analysis of existing de-identified data it did not require the board’s additional approval.

From fall 2014 through spring 2015, middle school and high school students completed paper-and-pencil questionnaires (82 and 84 questions, respectively) on various tobacco products: cigarettes, cigars, smokeless tobacco, pipe tobacco, bidis/kreteks, hookah, e-cigarettes, and some other new tobacco products. Current use for each tobacco product was defined as use on at least 1 day during the past 30 days. “Any tobacco product use” was defined as current use of any tobacco product listed. “Polyuse” was defined as current use of 2 or more tobacco products listed.

Weighted and design-corrected proportions and 95% confidence intervals (CIs) were calculated by using SAS SURVEY procedures (PROC SURVEYFREQ) of SAS 9.4 (SAS Institute, Inc). Rao–Scott χ^2^ tests (*F* values) were used to analyze relationships between product use and demographic characteristics. Sample sizes varied because of missing data for some characteristics or other variables. A significance level of .05 was used for performing and interpreting all analyses.

## Results

A total of 51.3% (n = 1,307) of middle school students and 51.1% (n = 1,049) of high school students were male (Table 1). Both samples were predominantly non-Hispanic white (middle school, 65.7% [n = 1,605]; high school, 68.5% [n = 1,453]) or non-Hispanic black (middle school, 13.9% [n = 285]; high school, 13.5% [n = 119]); only 7.6% (n = 288) of middle school and 6.5% (n = 160) of high school students were Hispanic, and 12.9% (n = 340) of middle school and 11.5% (n = 213) of high school students reported other non-Hispanic races (Table 2).

**Table 1:** Percentage of Middle and High School Students Who Currently^a^ Use Tobacco Products, Total and By Sex, Pennsylvania Youth Tobacco Survey, 2014–2015

Tobacco Product	No. of Respondents^b^	Total, % (95% CI)	Sex		
			Female, % (95% CI)	Male, % (95% CI)	*P *Value^c^
**High school students^d^**					
Electronic cigarettes	1,992	9.8 (7.5–12.0)	9.5 (6.9–12.2)	10.1 (7.0–13.1)	.75
Cigarettes	1,970	11.0 (8.1–13.8)	9.7 (6.7–12.6)	12.2 (8.3–16.1)	.18
Cigars	1,983	9.1 (7.0–11.2)	5.1 (3.2–7.0)	12.9 (9.7–16.0)	<.001
Hookah	1,992	5.0 (3.3–6.7)	5.7 (3.3–8.0)	4.4 (2.7–6.1)	.23
Smokeless tobacco	1,987	7.8 (5.9–9.7)	3.3 (1.5–5.1)	12.1 (9.3–14.9)	<.001
Pipe tobacco	2,003	3.7 (2.4–4.9)	3.1 (2.0–4.1)	4.1 (2.2–6.0)	.23
Bidis/kreteks	2,004	1.4 (0.9–1.8)	0.9 (0.4–1.5)^e^	1.8 (1.1–2.4)	.08
Other^f^	1,992	1.8 (1.2–2.4)	0.8 (0.1–1.4)^e^	2.7 (1.6–3.9)	.01
Any tobacco product use^g^	1,945	22.4 (18.3–26.5)	18.6 (14.1–23.2)	26.0 (21.1–30.8)	<.003
Polyuse^h^	1,945	12.4 (9.5–15.3)	9.2 (6.3–12.1)	15.4 (11.2–19.6)	.004
Total	—	—	48.9 (45.8–51.9)	51.1 (48.1–54.2)	—
**Middle school students^i^**					
Electronic cigarettes	2,609	2.3 (1.4–3.2)	2.4 (1.1–3.7)	2.2 (1.3–3.1)	.77
Cigarettes	2,576	1.9 (1.1–2.7)	2.5 (1.3–3.7)	1.3 (0.6–2.1)	.08
Cigars	2,586	0.8 (0.5–1.2)	0.7 (0.1–1.2)^e^	1.0 (0.4–1.7)^e^	.46
Hookah	2,609	0.9 (0.5–1.3)	0.8 (0.2–1.4)^e^	1.0 (0.5–1.5)	.67
Smokeless tobacco	2,592	1.2 (0.5–1.9)	0.8 (0.0–1.6)^e^	1.5 (0.6–2.5)^e^	.23
Pipe tobacco	2,632	1.3 (0.7–1.9)	1.1 (0.4–1.9)^e^	1.4 (0.7–2.1)	.42
Bidis/kreteks	2,644	0.4 (0.2–0.6)	0.5 (0.1–0.9)^e^	0.3 (0.0–0.6)^e^	.42
Other^f^	2,609	0.4 (0.1–0.6)^e^	0.3 (0.0–0.6)^e^	0.4 (0.1–0.7)^e^	.67
Any tobacco product use^g^	2,531	4.3 (3.0–5.6)	4.5 (2.8–6.3)	4.1 (2.6–5.6)	.68
Polyuse^h^	2,531	2.0 (1.3–2.7)	2.3 (1.2–3.5)	1.7 (1.0–2.4)	.24
Total	—	—	48.7 (46.3–51.0)	51.3 (49.0–53.7)	—

Abbreviations: —, not applicable; CI, confidence interval.

a “Current use” defined as use on ≥1 day in the past 30 days.

b Number of participants included in analysis; some participants were excluded from analysis because of missing values. Participants were 2,668 students from 72 middle schools and 2,017 students from 63 high schools.

c Rao–Scott χ^2^ test used to evaluate associations between reported product use and demographic characteristics.

d 2,008 high school students answered question on sex.

e Estimates should be interpreted with caution because relative standard error >30%.

f “Other” defined as some other new tobacco product.

g “Any tobacco product use” defined as current use of cigarettes, cigars, smokeless tobacco, electronic cigarettes, hookahs, pipe tobacco, bidis/kreteks, or some other new tobacco product.

h “Polyuse” defined as current use of 2 or more of the products listed in the table.

i 2,656 middle school students answered the question on sex.

**Table 2:** Percentage of Middle and High School Students Who Currently^a^ Use Tobacco Products, By Race/Ethnicity, Pennsylvania Youth Tobacco Survey, 2014–2015

Tobacco Product	No. of Respondents^b^	Race/Ethnicity				
		Non-Hispanic White, % (95% CI)	Non-Hispanic Black, % (95% CI)	Hispanic, % (95% CI)	Non-Hispanic Other, % (95% CI)	*P* Value^c^
**High school students^d^**						
Electronic cigarettes	1,992	11.7 (9.1–14.3)	4.7 (0.0–11.1)^e^	7.3 (3.3–11.4)	7.0 (3.4–10.5)	.09
Cigarettes	1,970	12.6 (9.0–16.3)	3.7 (0.9–6.4)^e^	11.8 (6.9–16.6)	9.2 (4.5–13.9)	.001
Cigars	1,983	11.0 (8.2–13.8)	3.5 (0.2–6.7)^e^	8.2 (3.4–12.9)	6.0 (2.6–9.5)	.005
Hookah	1,992	4.8 (3.0–6.6)	5.9 (0.0–12.2)^e^	7.2 (2.9–11.6)	2.9 (0.0–5.8)^e^	.61
Smokeless tobacco	1,987	9.3 (7.2–11.4)	4.3 (0.0–10.8)^e^	8.6 (3.8–13.4)	4.0 (1.2–6.8)^e^	.18
Pipe tobacco	2,003	3.8 (2.2–5.3)	0.8 (0.0–1.9)^e^	8.3 (4.4–12.3)	3.6 (0.4–6.9)^e^	.007
Bidis/kreteks	2,004	1.3 (0.7–1.9)	0.8 (0.0–1.9)^e^	5.1 (1.4–8.8)^e^	0.7 (0.0–1.7)^e^	.002
Other^f^	1,992	2.0 (1.1–2.8)	—g	2.9 (0.2–5.5)^e^	1.6 (0.0–3.3)^e^	—h
Any tobacco product use^i^	1,945	25.6 (20.7–30.4)	9.8 (2.8–16.8)^e^	22.8 (15.4–30.2)	16.6 (10.3–23.0)	<.001
Polyuse^j^	1,945	14.8 (11.3–18.3)	4.5 (0.0–11.1)^e^	11.4 (6.5–16.4)	8.5 (4.6–12.4)	.02
Total	—	68.5 (62.7–74.4)	13.5 (8.8–18.2)	6.5 (4.5–8.6)	11.5 (8.9–14.0)	—
**Middle school students^k^**						
Electronic cigarettes	2,609	2.6 (1.4–3.8)	1.9 (0.0–4.3)^e^	1.7 (0.3–3.0)^e^	2.3 (0.0–5.2)^e^	.91
Cigarettes	2,576	1.6 (0.8–2.5)	1.8 (0.2–3.4)^e^	2.2 (0.6–3.8)^e^	2.7 (0.0–5.7)^e^	.70
Cigars	2,586	0.7 (0.3–1.1)	0.9 (0.0–2.0)^e^	2.6 (0.8–4.5)^e^	—g	—h
Hookah	2,609	0.6 (0.2–1.0)^e^	1.0 (0.0–2.6)^e^	4.2 (0.9–7.6)^e^	0.4 (0.0–1.1)^e^	.001
Smokeless tobacco	2,592	1.2 (0.5–1.9)	0.4 (0.0–1.1)^e^	2.2 (0.6–3.8)^e^	1.6 (0.0–4.5)^e^	.49
Pipe tobacco	2,632	1.0 (0.5–1.6)	2.1 (0.0–4.9)^e^	3.2 (0.9–5.5)^e^	0.7 (0.0–1.7)^e^	.12
Bidis/kreteks	2,644	0.5 (0.2–0.7)	0.3 (0.0–1.0)^e^	0.3 (0.0–1.0)^e^	—g	—h
Other^f^	2,609	0.3 (0.0–0.6)^e^	—g	1.3 (0.0–2.7)^e^	0.6 (0.0–1.5)^e^	—h
Any tobacco product use^i^	2,531	3.9 (2.3–5.4)	4.3 (0.6–8.0)^e^	7.4 (4.0–10.7)	4.2 (1.1–7.4)^e^	.41
Polyuse^j^	2,531	1.7 (1.0–2.4)	2.1 (0.0–4.3)^e^	4.9 (2.4–7.5)	2.1 (0.0–5.2)^e^	.18
Total	—	65.7 (58.8–72.5)	13.9 (8.6–19.1)	7.6 (5.4–9.7)	12.9 (10.8–14.9)	—

Abbreviations: —, not applicable; CI, confidence interval.

a “Current use” defined as use on ≥1 day in the past 30 days.

b Number of participants included in analysis; some participants were excluded from analysis because of missing values. Participants were 2,668 students from 72 middle schools and 2,017 students from 63 high schools.

c Rao–Scott χ^2^ test used to evaluate associations between reported product use and demographic characteristics.

d 1,945 high school students answered the question on race/ethnicity.

e Estimates should be interpreted with caution because relative standard error is >30%.

f “Other” defined as some other new tobacco product.

g Zero frequency.

h Statistical results could not be generated because of zero frequency.

i “Any tobacco product use” defined as current use of cigarettes, cigars, smokeless tobacco, electronic cigarettes, hookahs, pipe tobacco, bidis/kreteks, or some other new tobacco product.

j “Polyuse” defined as current use of 2 or more of the products listed in the table.

k 2,518 middle school students answered the question on race/ethnicity.

Overall, 4.3% (95% CI, 3.0%–5.6%) of middle school students and 22.4% (95% CI, 18.3%–26.5%) of high school students reported current use of any tobacco product (Table 1). We found 2.0% (95% CI, 1.3%–2.7%) of middle school students and 12.4% (95% CI, 9.5%–15.3%) of high school students currently used 2 or more tobacco products. Among both middle school and high school students, e-cigarettes and cigarettes were the most commonly used tobacco products. Cigarettes were slightly more popular than e-cigarettes among high school students (11.0% vs 9.8%), and e-cigarettes were slightly more popular than cigarettes among middle school students (2.3% vs 1.9%).

A larger proportion of high school boys than girls reported use of cigars, smokeless tobacco, other new tobacco products, any tobacco product use, and polyuse (Table 1). Additionally, use of cigarettes, cigars, pipe tobacco, bidis/kreteks, any tobacco use, and polyuse had a significant association with race/ethnicity; however, small sample sizes should be considered in the interpretation of these results. We observed an increase in product use by grade for e-cigarettes, cigars, and hookah; trends were less clearly demonstrated for cigarettes, pipe tobacco, smokeless tobacco, bidis/kreteks, and other new tobacco products (Figure).

**Figure:**
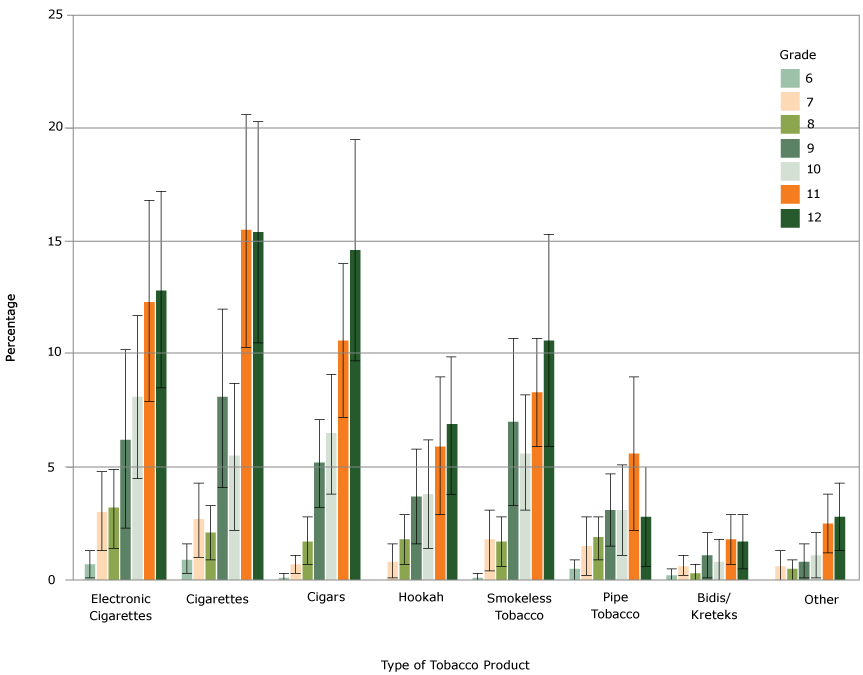
Percentages of middle and high school students who currently use tobacco, by grade and type of tobacco product, Pennsylvania Youth Tobacco Survey, 2014–2015. Error bars indicate 95% confidence intervals. Current use is defined as use on ≥1 day in the past 30 days. **Table:** 

Product Type	Middle School Grade Level, % (95% Confidence Interval)			High School Grade Level, % (95% Confidence Interval)			
	6	7	8	9	10	11	12
Electronic cigarettes	0.7 (0.1–1.3)	3.0 (1.3–4.8)	3.2 (1.4–4.9)	6.2 (2.3–10.2)	8.1 (4.5–11.7)	12.3 (7.9–16.8)	12.8 (8.5–17.2)
Cigarettes	0.9 (0.3–1.6)	2.7 (1.0–4.3)	2.1 (0.9–3.3)	8.1 (4.1–12.0)	5.5 (2.2–8.7)	15.5 (10.3–20.6)	15.4 (10.5–20.3)
Cigars	0.1 (0.0–0.3)	0.7 (0.3–1.1)	1.7 (0.7–2.8)	5.2 (3.2–7.1)	6.5 (3.8–9.1)	10.6 (7.2–14.0)	14.6 (9.7–19.5)
Hookah	0	0.8 (0.1–1.6)	1.8 (0.7–2.9)	3.7 (1.6–5.8)	3.8 (1.4–6.2)	5.9 (2.9–9.0)	6.9 (3.5–10.4)
Smokeless tobacco	0.1 (0.0–0.3)	1.8 (0.4–3.1)	1.7 (0.6–2.8)	7.0 (3.3–10.7)	5.6 (3.1–8.2)	8.3 (5.9–10.7)	10.6 (5.9–15.3)
Pipe tobacco	0.5 (0.0–0.9)	1.5 (0.2–2.8)	1.9 (0.9–2.8)	3.1 (1.5–4.7)	3.1 (1.1–5.1)	5.6 (2.2–9.0)	2.8 (0.6–5.0)
Bidis/kreteks	0.2 (0.0–0.5)	0.6 (0.2–1.1)	0.3 (0.0–0.7)	1.1 (0.1–2.1)	0.8 (0.0–1.8)	1.8 (0.7–2.9)	1.7 (0.5–2.9)
Other	0	0.6 (0.0–1.3)	0.5 (0.0–0.9)	0.8 (0.1–1.6)	1.1 (0.1–2.1)	2.5 (1.2–3.8)	2.8 (1.3–4.3)

## Discussion

During the 2014–2015 YTS period, 4.3% of middle school students and 22.4% of high school students were current users of at least one tobacco product, with 2.0% of middle school and 12.4% of high school students using multiple tobacco products. Cigarette use was slightly more common than e-cigarette use among high school students, in contrast to national estimates, which show e-cigarette use to be more common. The greater popularity of cigarettes among high school students may be due in part to Pennsylvania’s mostly rural population, whose adolescents prefer cigarettes over e-cigarettes (4). Another explanation may be that e-cigarette prevalence was underestimated by the YTS’s check-all-that-apply questions. This style of question may compel participants to skip answer choices even if they use a particular product (5).

Our study is a model for the monitoring, analysis, and reporting of data on tobacco use among adolescents. It provides a baseline to examine the effects of changes in state tobacco taxes and any new federal regulations under the purview of the US Food and Drug Administration (6). In 2016, Pennsylvania cigarette taxes increased from $1.60 to $2.60 per pack of 20 cigarettes, and a new tax was passed on e-cigarettes amounting to 40% of value (7). The new taxes on cigarettes and e-cigarettes may cause young people and adults to migrate from more heavily taxed products (now e-cigarettes and cigarettes) to less heavily taxed products (cigars). Pennsylvania does not tax cigars weighing more than 4 pounds per thousand (8). In Pennsylvania, the sale of tobacco products to minors (under age 18 y) has been prohibited since 2002 (9); however, the law’s current definition of tobacco products does not include e-cigarettes (9).

This survey was a representative sample of students who used a broad range of tobacco products. However, results are generalizable only to public school students in Pennsylvania. Data were self-reported and subject to recall and response bias. Despite these limitations, the prevalence estimates were relatable to those reported in national estimates.
